# Photorespiration and Carbon Limitation Determine Productivity in Temperate Seagrasses

**DOI:** 10.1371/journal.pone.0083804

**Published:** 2013-12-20

**Authors:** Pimchanok Buapet, Lina M. Rasmusson, Martin Gullström, Mats Björk

**Affiliations:** 1 Department of Ecology, Environment and Plant Sciences, Stockholm University, Stockholm, Sweden; 2 Department of Biology, Prince of Songkla University, Songkhla, Thailand; Dauphin Island Sea Lab, United States of America

## Abstract

The gross primary productivity of two seagrasses, *Zostera marina* and *Ruppia maritima*, and one green macroalga, *Ulva intestinalis*, was assessed in laboratory and field experiments to determine whether the photorespiratory pathway operates at a substantial level in these macrophytes and to what extent it is enhanced by naturally occurring shifts in dissolved inorganic carbon (DIC) and O_2_ in dense vegetation. To achieve these conditions in laboratory experiments, seawater was incubated with *U. intestinalis* in light to obtain a range of higher pH and O_2_ levels and lower DIC levels. Gross photosynthetic O_2_ evolution was then measured in this pretreated seawater (pH, 7.8–9.8; high to low DIC:O_2_ ratio) at both natural and low O_2_ concentrations (adjusted by N_2_ bubbling). The presence of photorespiration was indicated by a lower gross O_2_ evolution rate under natural O_2_ conditions than when O_2_ was reduced. In all three macrophytes, gross photosynthetic rates were negatively affected by higher pH and lower DIC. However, while both seagrasses exhibited significant photorespiratory activity at increasing pH values, the macroalga *U. intestinalis* exhibited no such activity. Rates of seagrass photosynthesis were then assessed in seawater collected from the natural habitats (i.e., shallow bays characterized by high macrophyte cover and by low DIC and high pH during daytime) and compared with open baymouth water conditions (where seawater DIC is in equilibrium with air, normal DIC, and pH). The gross photosynthetic rates of both seagrasses were significantly higher when incubated in the baymouth water, indicating that these grasses can be significantly carbon limited in shallow bays. Photorespiration was also detected in both seagrasses under shallow bay water conditions. Our findings indicate that natural carbon limitations caused by high community photosynthesis can enhance photorespiration and cause a significant decline in seagrass primary production in shallow waters.

## Introduction

In addition to CO_2_, marine plants can utilize HCO_3_^−^ from seawater as a source of carbon by various means [1], [2], [3], [4], [5], [6], [7]. The ability to utilize HCO_3_^−^ also allows the plants to maintain high internal CO_2_ concentrations, optimizing their photosynthetic capacity in an environment where the CO_2_ supply is often limited [8], [9], [10], [11] and where HCO_3_^−^ is approximately 150 times more abundant than CO_2_ [12]. The efficiency and mechanisms underlying this ability to utilize HCO_3_^−^ have been demonstrated to differ greatly between marine macrophyte species [5], [7], [9], [13], [14].

Rubisco is the key enzyme for primary CO_2_ fixation in all photolithotrophs as well as chemolithotrophs. In C3-type fixation it catalyses not only the carboxylation of ribulose bisphosphate (RuBP) in photosynthetic carbon assimilation but also its oxygenation. The process in which RuBP is oxygenated is called photorespiration and is generally favoured by low CO_2_ and high O_2_ levels [15]. It is considered a wasteful process because it lowers the efficiency of photosynthesis by competing with carbon fixation while consuming internal energy in the form of ATP and reducing equivalents. Photorespiration reportedly occurs in some species of seagrass (e.g., *Cymodocea rotundata* and *Halophila ovata*) and in a few other marine macrophytes [16], [17], [18], [19], [20], but its ecological significance has not been extensively studied. Although photorespiration seems to be possible in many marine plants, an efficient HCO_3_^−^ utilization system may suppress the oxygenase activity of Rubisco by supplying the catalytic site with an elevated level of CO_2_ [2], [21], [22]. In addition, the rates of photorespiration in seagrasses are apparently lower than in terrestrial plants [23], [24]. Hence, previous findings suggest that the photorespiratory process does not significantly reduce seagrass primary productivity.

Dissolved inorganic carbon (DIC) availability in shallow coastal waters often fluctuates greatly due to biological activities. While community respiration releases CO_2_ into the surrounding water and lowers seawater pH, photosynthetic carbon assimilation removes CO_2_ and increases the pH. A balance between these two simultaneous processes partly shapes the daily and seasonal variations of seawater carbonate chemistry. High pH brought about by high photosynthetic activity exceeding respiration during the day has been observed in many shallow coastal areas, i.e., seagrass meadows and macroalgae belts [25], [26], [27], [28], and also at a broader spatial scale corresponding to the area of an embayment [29]. In such areas where community photosynthesis is high, species with inferior carbon acquisition efficiency could be negatively affected as their carbon source (both CO_2_ and HCO_3_^−^) becomes more limiting for photosynthetic carbon fixation [30], [31]. Moreover, a low CO_2_:O_2_ ratio might enhance the oxygenation of RuBP in Rubisco, resulting in a more severe loss of plant productivity through photorespiration.

This study aimed to assess whether photorespiration caused any significant loss in the primary production of two common seagrasses, *Zostera marina* and *Ruppia maritima*, and a green macroalga, *Ulva intestinalis*, and to determine whether the contribution of photorespiration could be enhanced by the natural shifts towards higher pH and O_2_ brought about by high community photosynthesis in shallow coastal waters.

## Materials and Methods

### Ethics statement

No specific permits were required for the field sampling and the field studies did not involve endangered or protected species.

### Laboratory experiments

Representative specimens of *Zostera marina*, *Ruppia maritima*, and *Ulva intestinalis* were collected from two bays in the outer part of Gullmarsfjorden on the Swedish Skagerrak coast (58°20′–58°30′ N, 11°40′–11°50′ E). All specimens were immediately transported to the laboratory, where they were kept in dim light and running natural seawater before use in experiments on the same day.

**Figure 1:**
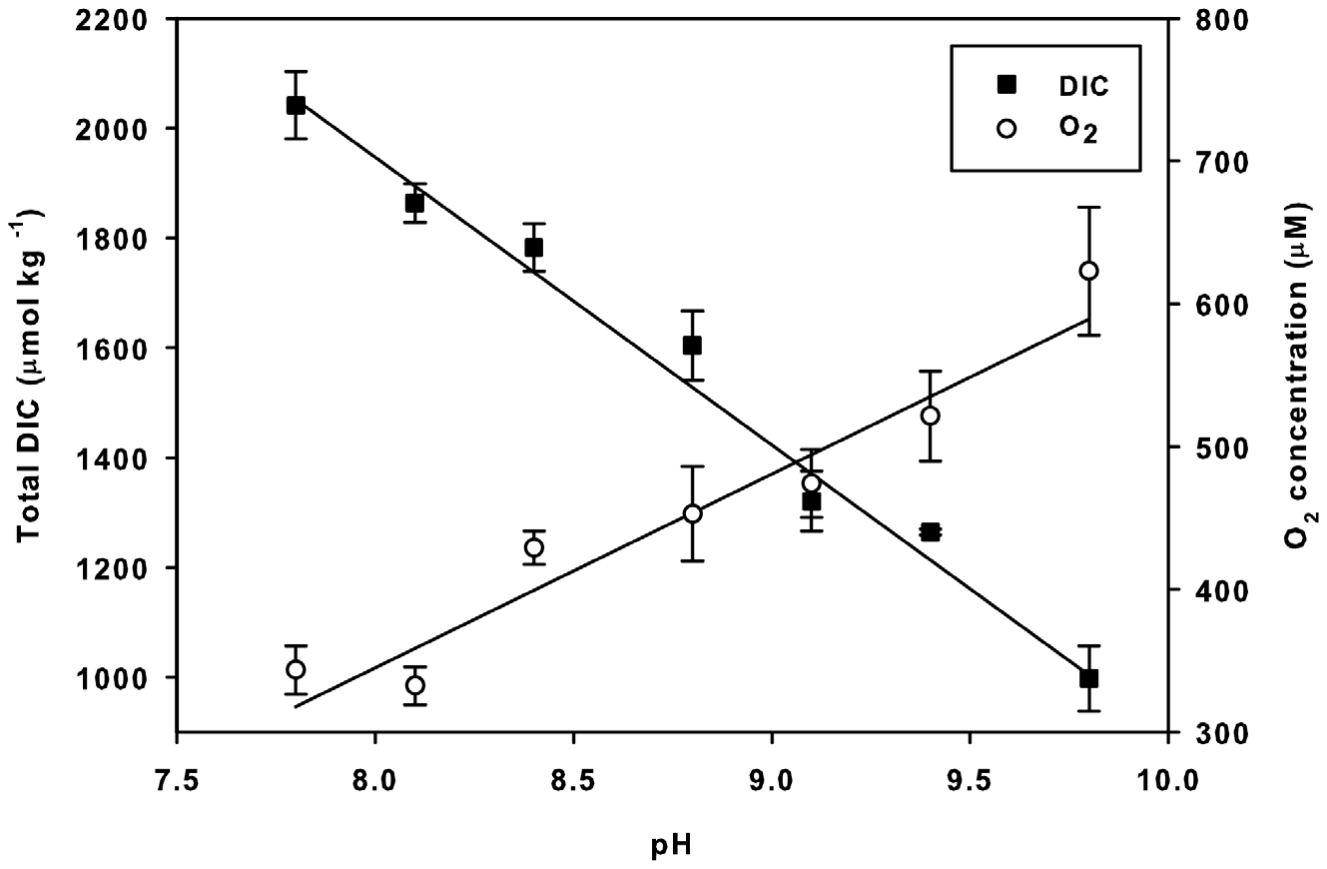
Total seawater DIC and O_2_ contents at the pH values used in the experiments.

To obtain ecologically relevant experimental conditions with different pH, DIC, and O_2_ levels, natural seawater (salinity 23–25) was incubated in 1.5-L glass bottles with *U. intestinalis* and kept in sunlight before the experiments. The photosynthetic carbon assimilation caused a decrease in DIC and subsequently a rise in pH, while photosynthetic oxygen evolution simultaneously caused a rise in O_2_ tension. In this way, the obtained pH (measured using a model 340i Multimeter; WTW, Weilheim, Germany) varied from 7.8 to 9.8 (Figure 1). From each run, three 4-mL subsamples of seawater were taken for measurements of DIC and total alkalinity (TA), while the pH was recorded immediately before and after adding 1 mL of 0.01 M HCl. The alkalinity was determined according to the rapid electrometric determination method described by Anderson and Robinson [32] and downscaled according to Semesi *et al.* [33]. The total inorganic carbon of the water was calculated according to Riley and Skirrow [34] using constants obtained from Smith and Kinsey [35]. The average pH and TA values were used in calculating the DIC concentration with the computer program CO_2_sys.xls (ver.10) [36].

Oxygen flux measurements were used to determine the gross photosynthetic oxygen evolution rates of the studied plants. The measurements were made in three 3-mL incubation chambers connected to Clark-type oxygen electrodes (DW1/AD; Hansatech Instruments, King's Lynn, UK); the temperature was kept constant at 20°C. Segments of seagrass leaves (either one piece of *Z. marina* 3 cm long and 0.3–0.5 cm wide or five pieces of *R. maritima* 3 cm long and 0.1 cm wide) were fixed in a U-shape in the chamber, while in the experiments with *U. intestinalis*, a piece 3 cm long and 0.3–0.5 cm wide was left floating freely in the chamber. Pretreated seawater (2.4 mL) was added to the chambers and constantly stirred by a magnetic stirrer. Once the initial oxygen level was recorded, the samples were subjected to 10–15 min of darkness to obtain dark respiration rates. Then the water was changed and actinic light was provided using a cold light source (KL 1500 LCD; Zeiss, Oberkochen, Germany) at an intensity of 400 µmol photons m^−2^ s^−1^ to obtain the net photosynthetic rate. It took approximately 10–15 min to reach steady-state photosynthesis, at which point the net oxygen evolution rate at a normal O_2_ level was recorded. The water was then bubbled with N_2_ to reduce the O_2_ concentration to 50 µM (i.e., approximately 20% of the natural concentration, when in equilibrium with air); the dark respiration rate was once again measured, followed by the net photosynthesis. (The effect of N_2_ purging on dissolved CO_2_, and thus also pH, was also tested and found to be less than 10% for free CO_2_, and accordingly negligible on total DIC). The measurements were also made in a reversed order starting with low O_2_. The same procedure was repeated using new seagrass and fresh seawater incubated with *U. intestinalis* (as previously described). Since manipulating the O_2_ level could affect the respiratory O_2_ consumption rates in seagrass [37], dark respiration rates were measured under both natural and low-O_2_ conditions. Gross photosynthetic rates were calculated as the net photosynthetic rates + dark respiration rates.

As an additional control, the O_2_ flux in chambers without plants but filled with either distilled or *Ulva*-incubated water was measured following the same steps as used with plants. Neither oxygen consumption in the dark nor oxygen release in the light was observed. Moreover, after the oxygen level in the chambers had been reduced by N_2_ bubbling, there was no rise in the O_2_ concentration, so an increase of O_2_ through leakage could be ruled out.

Furthermore, plant samples measured at pH 9.8 were re-measured after purging the water with N_2_+1% CO_2_ (eliminating O_2_ and providing saturating CO_2_). As this caused a recovery of gross photosynthetic rates, the photosynthetic response was assumed to be affected predominantly by the DIC availability and O_2_ level.

Photorespiration competes with carbon assimilation, resulting in a lowered photosynthetic rate. However, the process also consumes oxygen, so decreased gross oxygen evolution rates are expected under photorespiratory conditions. For these reasons, a lower gross photosynthetic rate under normal conditions (i.e., natural O_2_) than under O_2_-depleted conditions was used as a measure of photorespiration in these studies. The relative level of photorespiration at each pH was defined as the percentage reduction of the gross photosynthetic rate under photorespiratory conditions (natural O_2_ concentration) versus non-photorespiratory conditions (low O_2_).

### Experiments with field-collected seawater

Two seagrasses, *Zostera marina* and *Ruppia maritima*, and seawater were collected on two clear days from inside two sheltered bays (i.e., Skallhavet and Fiskebäckskil, both considered appropriate bays sensu Gullström *et al.* [38]) during hours of high photosynthetic activity (12:00–15:00 h) and immediately transported to the laboratory. Seawater was collected from two parts of the bays: the shallow area in the bays inhabited by seagrass and other macrophytes (bay water, low DIC and high pH) and the entrance of the bays (baymouth water, where water DIC was in equilibrium with air). There were two different setups. The first setup was designed to investigate whether seagrass growing in the shallow bay was carbon limited by comparing the gross photosynthetic rates measured in bay water with the rates measured in the baymouth open water. To confirm that the observed changes in photosynthesis were due to carbon limitation, the photosynthetic rates were also measured in bay water purged with CO_2_ until the pH of the baymouth water was reached, thus restoring DIC levels (at approximately pH 8.1). The second setup focused on assessing seagrass photorespiration in the shallow bay by comparing gross photosynthetic rates in bay water at normal and low O_2_ levels. The measurements followed the same procedures described in the previous section. The ambient light intensity of approximately 400 µmol photons m^−2^ s^−1^ was used for incubation (measured using a DIVING-PAM underwater chlorophyll fluorometer; Waltz, Effeltrich, Germany). A refractometer was used to measure the salinity, which was 25–28, and the seawater temperature was 24–25°C. TA and DIC were determined using the same methods described previously.

### Statistical analysis

#### Laboratory experiments

Regression analysis was conducted to describe the relationships between total DIC and O_2_ concentrations and pH value, respectively. In further analyses, pH was used as a proxy for water chemistry variables. The effect of O_2_ condition (normal or low O_2_) and pH on the photosynthetic rates of the three macrophytes was tested using repeated-measures ANOVA (O_2_ condition as the within-group factor and pH as the categorical factor). Fisher's least significant difference (LSD) test was used to determine the pH value at which the gross photosynthesis differed between the normal and low-O_2_ conditions. Functional relationships between pH and relative gross photosynthetic rates (percentage of the rates at the reference pH 8.1, i.e., the pH of the seawater well equilibrated with air) in all three macrophytes were calculated using linear regression, and significant differences between the linear relationships were tested using an analysis of covariance (ANCOVA). The relationship between photorespiration level in both seagrasses (% reduction of photosynthetic rate under photorespiratory conditions, dependent variable) and pH (independent variable) were assessed using regression analysis. The relationships between dark respiration rates and the pH and initial O_2_ concentration of *Ulva*-incubated seawater were also assessed using regression analysis. The effect of the low-O_2_ treatment on the dark respiration rates of the three macrophytes was tested using repeated-measures ANOVA. Before conducting ANOVAs, the assumption of homogeneity of variances was tested using Cochran's Test.

#### Experiments with field-collected seawater

Differences in pH, TA, and O_2_ concentration were tested using nested ANOVAs with Site (two levels) and Waterbody (two levels, nested within Site) as fixed factors. Repeated-measures ANOVAs were conducted to analyse the effects of waterbody (baymouth water, bay water, and bay water purged with CO_2_) and O_2_ condition (normal and low O_2_) on seagrass photosynthetic rates (waterbody and O_2_ condition as within-group factors and site as the categorical factor). Before conducting ANOVAs, the assumption of homogeneity of variances was tested using Cochran's test.

## Results

### Laboratory experiments: loss in primary production with decreasing DIC, increasing pH, and increasing O_2_

During incubation with *U. intestinalis* in light, the seawater pH was raised from 7.8 to 9.8. Total DIC decreased, while O_2_ concentrations increased with increasing pH (regression analysis, *p*<0.05 for all measurements). Total DIC in the seawater was reduced from approximately 2100 µmol kg^−1^ to approximately 1000 µmol kg^−1^ (Figure 1). At the same time, the O_2_ concentration increased from approximately 300 µM to 700 µM (Figure 1).

**Figure 2:**
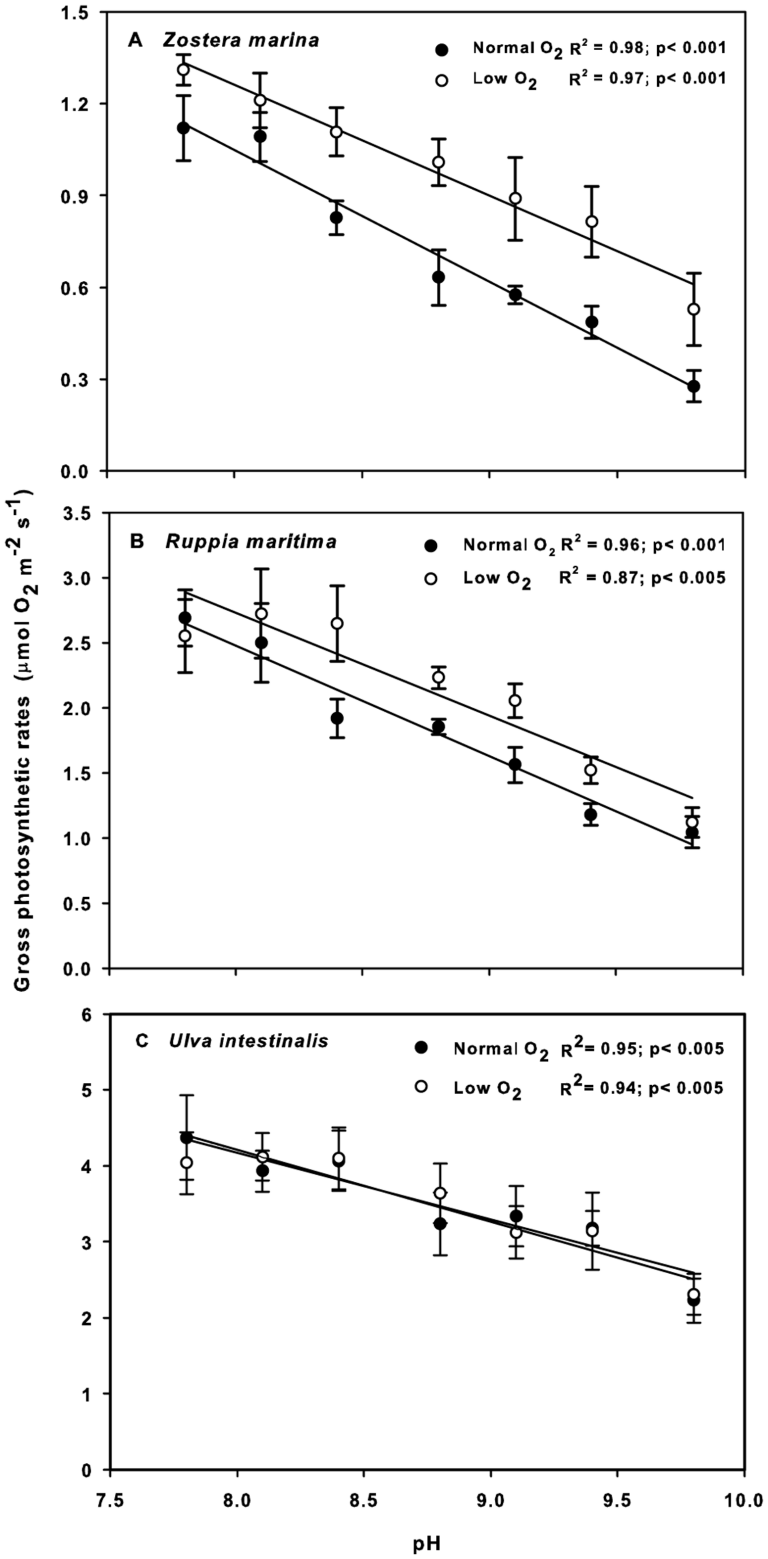
Gross photosynthetic rates as a function of pH under natural versus O_2_-depleted conditions. A) *Zostera marina*, B) *Ruppia maritima*, and C) *Ulva intestinalis*. See Figure 1 for Total DIC and O_2_ contents of seawater at each pH value.

**Figure 3:**
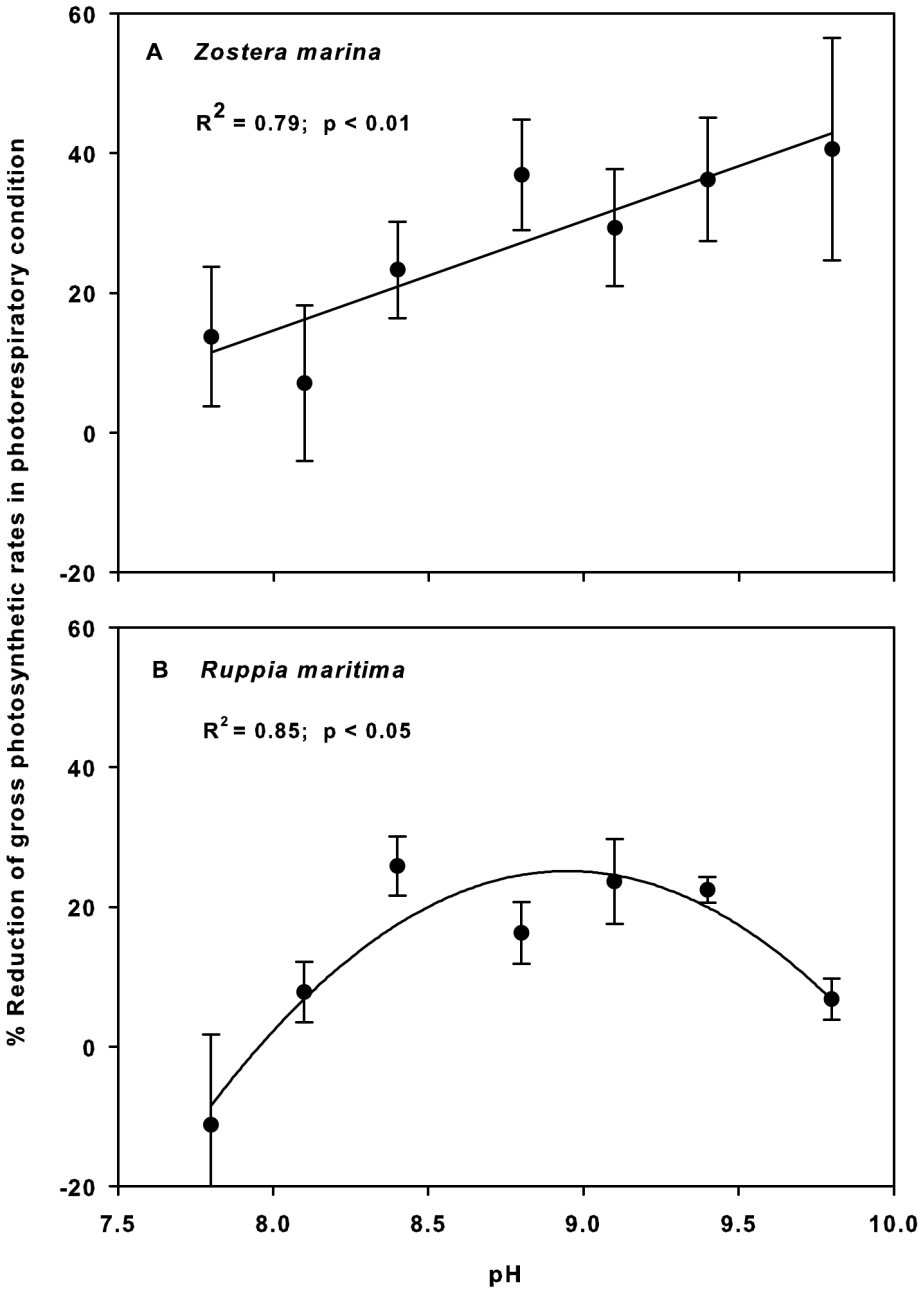
Percentage reduction of gross photosynthetic rates under photorespiratory conditions as a function of pH. A) *Zostera marina* and B) *Ruppia maritima*. The figure was produced from the data shown in Figure 2. *Ulva intestinalis* was excluded from regression analysis, as O_2_ conditions had no significant effect on its photosynthetic rates. See Figure 1 for DIC and O_2_ contents of seawater at each pH value.

Gross photosynthetic rates in all species were affected by increasing pH in both the natural O_2_ and low-O_2_ treatments (Figure 2). Under the normal O_2_ condition, the relative gross photosynthetic rates (percentage of the rates at pH 8.1) of the two seagrasses decreased more with increasing pH than did those of *U. intestinalis* (ANCOVA, *p*<0.001). For example, at pH 9.8, *Z. marina*, *R. maritima*, and *U. intestinalis* maintained 25%, 42%, and 57% of their gross photosynthetic rates, respectively. Under the low-O_2_ condition, no significant difference was detected between species. Effects of pH and O_2_ level were observed in both seagrasses (ANOVA, *p*<0.005 for all; Table 1), while no significant effect of O_2_ level was detected in *U. intestinalis*. A significant interaction between pH and O_2_ level was detected in *R. maritima* (ANOVA; *p*<0.05) but not in *Z. marina*. Fisher's LSD test revealed an O_2_-condition effect from pH 8.4 to 9.8 in *Z. marina* and from pH 8.8 to 9.1 in *R. maritima* (Figure 2). Regression analysis demonstrated a positive relationship between pH and photorespiration level (measured as the percentage reduction of photosynthetic rates under photorespiratory conditions) in *Z. marina*. Such a trend was less apparent in *R. maritima*, in which the photorespiration level increased up to pH 8.4, after which it levelled off and eventually decreased when pH reached 9.8 (Figure 3). We observed no significant effect of pH or O_2_ level on dark respiration in the range of the experimental conditions, i.e., pH 7.8–9.8 and O_2_ concentrations of 300–700 µM. However, the 50 µM low-O_2_ treatment lowered the dark respiration rates to 61±4.7%, 49±2.8%, and 44±11.7% of the rates at the normal O_2_ level in *Z. marina*, *R. maritima*, and *U. intestinalis*, respectively.

**Table 1:** Results of the repeated-measures ANOVA for net photosynthetic rates of *Zostera marina*, *Ruppia maritima*, and *Ulva intestinalis*.

	*Z. marina*		*R. maritima*		*U. intestinalis*	
Source of variation	F	P	F	P	F	P
pH	17.897	***p*** **<0.001**	10.469	***p*** **<0.001**	3.934	***p*** **<0.01**
O_2_ condition	36.217	***p*** **<0.001**	22.039	***p*** **<0.001**	0.022	*p* = 0.883
O_2_ condition × pH	0.545	*p* = 0.769	2.980	***p*** **<0.05**	0.720	*p* = 0.636

### Experiments with field-collected seawater: carbon limitation and photorespiration in natural settings

**Figure 4:**
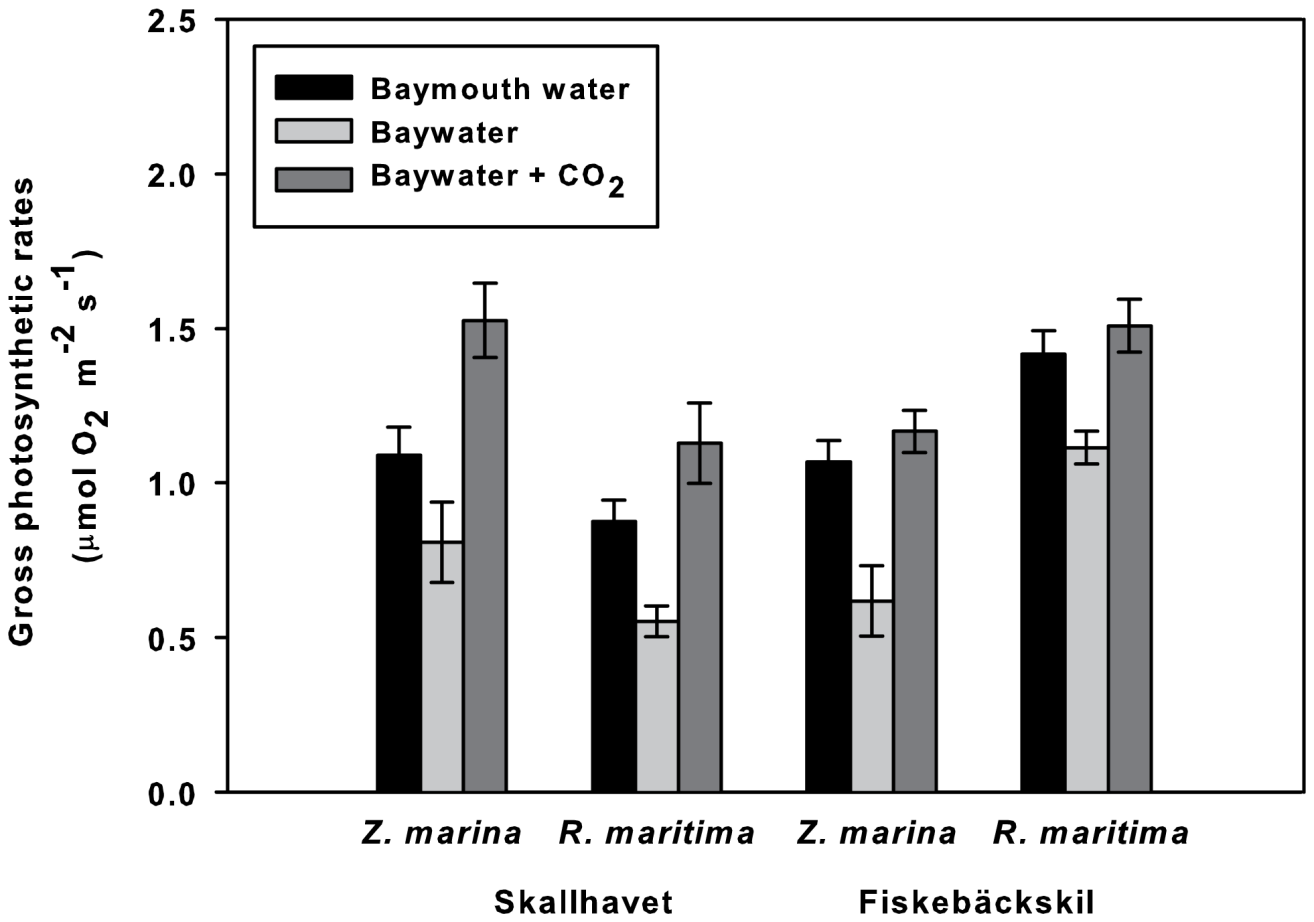
Gross photosynthetic rates of seagrasses in baymouth water, bay water, and CO_2_-purged bay water. *n* = 6; error bars indicate SE.

The pH values, DIC contents, and O_2_ concentrations at the field sampling sites are shown in Table 2. A significant difference in pH and TA was detected between bay water and baymouth water as well as between sites (ANOVA, *p*<0.05; Table 3). At both study sites, pH was higher and TA lower in bay water than in baymouth water (Table 2). Significant differences in O_2_ level were detected between sites but not between waterbodies. The gross photosynthetic rates of *Z. marina* and *R. maritima* in bay water were both lower than those in baymouth water (ANOVA, *p*<0.05; Figure 4), but the rates increased once the bay water was purged with CO_2_ until the pH of the baymouth water was reached. No significant difference in *Z. marina* photosynthesis was detected between sites, while a significant interaction between waterbody type and site was present in *R. maritima* (Table 4).

**Table 2:** A summary of pH and DIC components of baymouth water and bay water from both sampling sites, i.e., Skallhavet and Fiskebäckskil.

	Skallhavet		Fiskebäckskil	
Seawater chemistry	Baymouth water	Bay water	Baymouth water	Bay water
pH	8.08±0.01	8.71±0.01	8.06±0.01	8.47±0.02
TA (μmol kg^−1^)	1804.03±14.66	1757.85±30.17	1915.47±19.36	1822.06±7.39
Total DIC (μmol kg^−1^)	1684.78±13.59	1362.43±24.36	1694.69±9.97	1601.02±21.67
CO_2_ (μmol kg^−1^)	16.54±0.12	2.73±0.05	16.91±0.51	5.74±0.35
HCO_3_^−^ (μmol kg^−1^)	1575.77±12.50	1090.77±18.88	1581.78±10.90	1378.68±24.74
CO_3_^−^ (μmol kg^−1^)	92.47±1.10	268.93±5.75	95.99±1.66	216.59±7.18
O_2_ (μM)	292.83±12.16	269.33±12.33	302.83±17.80	337.16±8.93

**Table 3:** Comparisons of pH, total alkalinity, and O_2_ concentration in nested ANOVAs for both the Skallhavet and the Fiskebäckskil sites and in both waterbody types, i.e., bay and baymouth.

		pH		TA		O_2_	
Source of variation	Df	F	P	F	P	F	P
Site	1	2103122	***p*** **<0.001**	19.85	***p*** **<0.001**	8.697	***p*** **<0.01**
Waterbody (Site)	1	129	***p*** **<0.001**	6.99	***p*** **<0.01**	2.485	0.109

**Table 4:** Comparisons of gross photosynthetic rates of seagrasses measured in different types of waterbody, i.e., bay water, baymouth water and CO_2_-purged bay water for both the Skallhavet and the Fiskebäckskil sites in repeated-measures ANOVA.

	*Z. marina*		*R. maritima*	
Source of variation	F	P	F	P
Site	2.508	0.144	31.606	***p*** **<0.001**
Waterbody	44.343	***p*** **<0.001**	28.947	***p*** **<0.001**
Waterbody × Site	3.115	0.066	1.182	0.327

**Figure 5:**
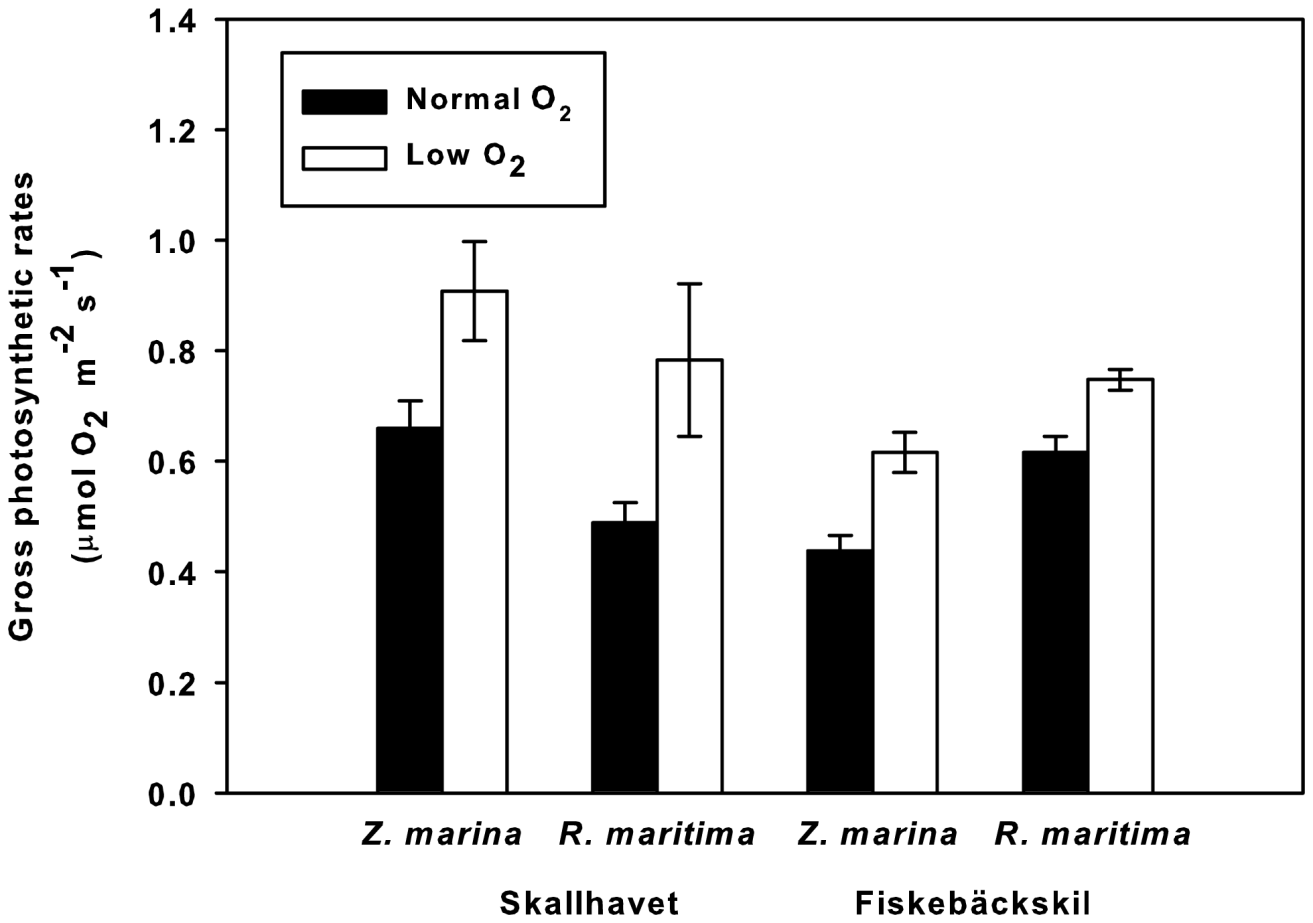
Gross photosynthetic rates of seagrasses under natural O_2_ concentrations versus O_2_-depleted conditions. *n* = 6; error bars indicate SE.

The gross photosynthetic rates of *Z. marina* and *R. maritima* under the normal O_2_ condition were lower than those in low O_2_ (ANOVA, *p*<0.05; Figure 5). A difference in *Z. marina* was also detected between sites (ANOVA; *p*<0.05), but there was no interaction between O_2_ condition and site. In the case of *R. maritima*, no significant difference between sites and no significant interaction between site and O_2_ condition were detected (Table 5).

**Table 5:** Comparisons of gross photosynthetic rates of seagrasses measured in bay water with two different O_2_ levels, i.e., natural and low O_2_ concentration for both the Skallhavet and the Fiskebäckskil sites in repeated-measures ANOVA.

	*Z. marina*		*R. maritima*	
Source of variation	F	P	F	P
Site	15.149	***p*** **<0.01**	0.324	0.582
O_2_ conditions	23.380	***P*** **<0.001**	10.438	***p*** **<0.01**
O_2_ conditions × Site	0.646	0.440	1.539	0.243

## Discussion

When pH increased, all three macrophytes displayed continuously decreasing photosynthetic rates, while only the two seagrasses exhibited photorespiratory activity. The decrease in photosynthesis at high pH was likely due to the decreasing availability of usable forms of DIC that occurs at higher pH values, while the total DIC level was sinking due to consumption [5], [25], [26], [39]. Together with decreasing gross photosynthetic rates, an O_2_-level effect was observed in the two seagrasses. This indicates that part of the loss in photosynthetic efficiency seen when pH increased can be explained by photorespiration. The O_2_ sensitivity effect was exhibited when pH exceeded 8.1, in line with the commonly accepted notion that when available CO_2_ at the active site of Rubisco decreases relative to O_2_ levels, the oxygenation of ribulose bisphosphate (RuBP) increases [40]. Unlike under “normal” O_2_ conditions, at which the macroalga performed better than did the two seagrasses, all three macrophytes responded similarly to increasing pH under non-photorespiratory conditions. This further supports the suggestion that photorespiration is the underlying cause of lower photosynthetic efficiency in the observed seagrasses, and that such a susceptibility to photorespiration is likely a result of limited capacity of their carbon acquisition mechanisms [5]. The O_2_ sensitivity of photosynthesis has been observed in other submerged aquatic macrophytes, including some seagrasses [2], [16], [41], [42], [43], and photorespiration has been proposed to cause a decline in photosynthesis in *Halophila stipulacea* when O_2_ accumulates under flow-restricted conditions [44]. The present study demonstrates that seagrass photorespiratory activity is induced by natural variations in the surrounding water caused by the primary productivity of other plants in the system. Moreover, as photosynthesis became more suppressed by low carbon availability, photorespiration increased greatly, particularly in *Z. marina*. Photorespiration could, under such conditions, reduce photosynthetic capacity with up to 40%. This highlights the ecological significance of photorespiration, especially in productive shallow coastal areas where DIC is usually limiting and the O_2_:DIC ratio is high. This was confirmed by the experiments conducted with field-collected seawater. The lower gross photosynthetic rates under natural O_2_ versus O_2_-depleted conditions indicated that photorespiration was taking place in seagrasses in their natural settings where DIC was limited. Although no difference in O_2_ concentration was observed between baymouth open water and bay water, DIC in the shallow bay was insufficient to eliminate the competitive effect of O_2_. We did not find any significant O_2_ effect on gross photosynthesis in *U. intestinalis*. However, this was to be expected, as various *Ulva* species are reportedly able to suppress photorespiration [45], [46] by maintaining an efficient carbon-concentrating mechanism (CCM) that supplies the active site of Rubisco with high CO_2_ levels and consequently suppresses the oxygenase activity [21], [47].

The presence of a more efficient CCM can also explain why the macroalga *U. intestinalis* was less affected by increasing pH than were the two seagrasses. A highly efficient CCM allows many macroalgae, such as *Ulva*, to thrive under high pH/low DIC conditions [27], [30], and by maintaining severe carbon limiting conditions by photosynthetic activity, *Ulva* could potentially exclude less efficient coexisting species [30].

The studies using field-collected seawater provide further evidence that DIC limits seagrass primary productivity in shallow habitats, consistent with the findings of several prior works [5], [7], [10], [11], [48], [49], [50], [51]. The lower photosynthetic rates of the seagrasses in the shallow bay than in the baymouth open water likely resulted from carbon limitation driven by the high photosynthetic activity of the shallow macrophyte community.

Also worth mentioning is the O_2_ effect on dark respiration. In this study we observed decreased dark respiration rates in low-O_2_ treatments, which contributed to increased net photosynthetic rates under O_2_-depleted conditions. Such O_2_-dependent dark respiration was previously observed in *Zostera marina* [37].

Overall, these results suggest that the common view that photorespiration rates are negligible in seagrasses might be untrue. Assessing seagrass primary productivity without considering losses through photorespiration might overestimate the production capacity, especially when using fluorescence parameters as a tool. Consequently, photorespiration should be taken into account when attempting to estimate the true carbon budgets of seagrass meadows in natural habitats where DIC fluctuates. However, while this study treats the photorespiratory process solely as a loss in seagrass primary productivity, photorespiration might also benefit the plant, as it might serve as an alternative energy sink for photosynthetic electron transport, protecting the plant from over-reduction of the electron transport chain and from photoinhibition [52], [53], [54], [55].
